# Nutritional Risk Profiles and Poor Self-Perceived Oral Health in Hungarian Adults: A Population-Based Study with Monte Carlo Population-Attributable Fraction Estimation

**DOI:** 10.3390/nu18152408

**Published:** 2026-07-23

**Authors:** Amr Sayed Ghanem, Battamir Ulambayar, Róbert Bata, Marianna Móré, Renáta Jávorné Erdei, Attila Csaba Nagy

**Affiliations:** 1 Department of Epidemiology, Faculty of Health Sciences, University of Debrecen, 4028 Debrecen, Hungary; aghanem@etk.unideb.hu (A.S.G.); ulambayar.battamir@etk.unideb.hu (B.U.); bata.robert@etk.unideb.hu (R.B.); 2 Institute of Social and Sociological Sciences, Faculty of Health Sciences, University of Debrecen, 4400 Nyíregyháza, Hungary; more.mariann@etk.unideb.hu; 3 Department of Health Methodology and Prevention, Faculty of Health Sciences, University of Debrecen, 4400 Nyíregyháza, Hungary; erdei.renata@etk.unideb.hu

**Keywords:** oral health, nutrition, diet, periodontitis, caries, gum disease, prevention

## Abstract

Background: Oral diseases remain a major public health challenge globally and in Hungary, where untreated caries, periodontal disease, and edentulism are highly prevalent. Diet is a key modifiable determinant of oral health, yet the cumulative impact of multiple unfavorable nutritional behaviors on self-perceived oral health has not been assessed at the national level in Hungary, nor have population-attributable fraction (PAF) estimates been reported. Methods: This cross-sectional study analyzed data from 5146 adults in the 2019 Hungarian European Health Interview Survey. A six-component nutritional-risk score was constructed from self-reported dietary behaviors (fruit intake, vegetable intake, sugary soft drink consumption, sweets consumption, processed meat consumption, and water intake). Survey-weighted logistic regression estimated associations between nutritional-risk categories and poor self-perceived oral health, adjusting for sociodemographic, lifestyle, and health-related covariates. Counterfactual PAFs with 95% Monte Carlo uncertainty intervals (1000 bootstrap replications) were estimated for three nutritional-risk scenarios. Results: The weighted prevalence of poor self-perceived oral health was 19.2%. Compared with participants with 0–1 unfavorable nutritional factors, those with 3 factors (OR = 1.37; 95% CI: 1.09–1.73) and 4–6 factors (OR = 1.46; 95% CI: 1.15–1.85) had significantly higher odds of poor oral health. Low water intake was independently associated with poor oral health (OR = 1.38; 95% CI: 1.16–1.64). The main counterfactual PAF analysis estimated that 16.1% (Monte Carlo 95% UI: 5.93–26.48%) of poor oral health burden was attributable to unfavorable nutritional profiles. High nutritional risk was also associated with active caries, gum bleeding, and tooth loss. Conclusions: Cumulative nutritional risk is independently associated with poor self-perceived oral health in Hungarian adults. Integrating dietary improvements into oral health prevention strategies may substantially reduce the population-level burden of oral disease.

## 1. Introduction

Oral diseases remain among the most common chronic health conditions worldwide and are now increasingly understood as a core non-communicable disease issue rather than a narrowly dental problem. The World Health Organization estimates that oral diseases affect nearly 3.7 billion people [1], and the 2021 Global Burden of Disease analysis reported that the combined age-standardized prevalence of the main oral conditions corresponded to 3.69 billion affected people globally, with untreated dental caries in permanent teeth remaining the single most common condition [2]. Importantly, this burden has changed little over the past three decades, indicating that past prevention and treatment approaches have not achieved meaningful population-level reductions. The WHO European regional summary similarly frames oral health as a continuing public-health issue across the 53-country Region, while broader conceptual work has emphasized that oral diseases are socially patterned, commercially shaped, and tightly linked to the wider non-communicable disease agenda [3]. This framing is now reflected in the renewed WHO oral-health strategy toward 2030, which explicitly embeds oral health within prevention, primary care, and universal health coverage [4,5].

This perspective is highly relevant to Hungary. Although the European Region showed smaller increases than several other WHO regions in the 1990–2021 period, stability at a high burden should not be mistaken for success [2]. In Hungary, the WHO oral health country profile estimated in 2019 a prevalence of 37.9% for untreated caries in permanent teeth, 8.6% for severe periodontal disease, and 14.1% for edentulism, while also documenting per-capita sugar availability of 55.1 g/day [6]. Hungarian studies further suggest that oral-health inequalities remain structured by age, education, financial status, and smoking, and that substantial caries experience and tooth loss continue to characterize the adult population [7,8]. Taken together, these data make Hungary a particularly relevant setting in which to study modifiable nutritional contributors to oral health disadvantage.

Diet is one of the most plausible and policy-relevant modifiable pathways. WHO guidance continues to identify free sugars as the principal dietary factor in the development of dental caries and recommends reducing intake to below 10% of total energy, with further reduction to below 5% conditionally recommended for additional benefit [9]. The epidemiologic evidence supporting this position has remained consistent over time: the systematic review that informed the WHO guideline, together with its ten-year update, found that lower sugars intake is associated with lower levels of dental caries [10,11,12]. More recent work also indicates that the diet–oral health relationship extends beyond caries alone. Systematic review evidence suggests that higher free-sugar intake is associated with periodontal diseases, while observational studies in large adult cohorts have shown that healthier dietary patterns and greater fruit and vegetable consumption are linked with lower odds of periodontitis and better tooth retention [13]. For a study focused on nutritional risk in oral health, this broader dietary framing is more appropriate than a caries-only narrative because it accommodates both biofilm-mediated and inflammation-related oral outcomes [14,15].

That broader framing is especially important in population surveys, where dietary behaviors cluster rather than occur in isolation. Individuals who consume fewer fruits and vegetables may also consume more sweets or sugar-sweetened drinks and follow less favorable overall dietary patterns; examining a single exposure at a time can therefore understate the cumulative burden of adverse nutrition-related behaviors [1]. Water intake also deserves attention in this context. WHO explicitly recommends favoring water as the main drink for oral-health prevention, and emerging epidemiologic evidence suggests that higher plain-water intake may be associated with lower odds of periodontitis [16]. A cumulative nutritional-risk profile may therefore capture public-healthly relevant exposure patterns more effectively than isolated dietary variables alone.

For population-based research, self-rated oral health is also a meaningful endpoint rather than merely a pragmatic substitute for clinical examination. Recent validation work has shown that poorer self-rated oral health tracks with more missing teeth, more untreated decay, and more severe periodontal attachment loss, supporting its use as a proxy measure of oral health status in survey settings [17]. In addition, self-reported oral-health measures have shown clinically meaningful associations with systemic comorbidity and all-cause mortality in large epidemiologic cohorts [18]. Accordingly, self-perceived oral health is well suited to national public-health surveillance because it captures the lived burden of oral disease while remaining feasible in large representative surveys.

An important gap nevertheless remains. In Hungary, the available literature has mainly described disease burden itself or broader sociodemographic and lifestyle correlates of oral health, but has not sufficiently clarified whether a combined nutritional-risk profile is independently associated with poor self-perceived oral health at the national level [7,8]. Based on the literature identified for this review, Hungarian analyses have also not yet translated such associations into policy-facing estimates of population burden using model-based population-attributable fractions [19]. That omission matters because odds ratios alone do not answer the more practical question of how much poor oral-health burden might theoretically be reduced under a healthier exposure distribution. Population-attributable fractions, particularly when estimated with regression-based counterfactual methods, provide that complementary perspective under explicit assumptions [20,21]. Accordingly, the present study used nationally representative data from the 2019 Hungarian European Health Interview Survey (EHIS) to examine the associations of individual dietary indicators and a composite nutritional-risk profile with poor self-perceived oral health in adults, and to estimate the population-attributable fraction of poor self-perceived oral health associated with unfavorable nutritional profiles. In addition, the study explored whether high nutritional risk was related to selected secondary oral-health outcomes, namely active caries, gum bleeding, tooth mobility, missing teeth, and difficulty eating.

## 2. Materials and Methods

### 2.1. Study Design and Data Source

This study was designed as a cross-sectional, population-based secondary analysis of the 2019 Hungarian EHIS [22]. The analysis focused on the adult Hungarian population and examined the association between nutritional-risk indicators and poor self-perceived oral health. The EHIS provides individual-level information on sociodemographic characteristics, health status, chronic conditions, lifestyle behaviors, healthcare utilization, oral-health indicators, and selected nutritional variables [23]. Because the dataset was derived from a population survey, all descriptive and regression analyses incorporated probability weights to improve representativeness of the Hungarian adult population [24].

The analytical objective was twofold. First, we examined whether individual nutritional indicators and a combined nutritional-risk profile were associated with poor self-perceived oral health. Second, we estimated the population-level burden of poor self-perceived oral health associated with unfavorable nutritional-risk profiles using counterfactual population-attributable fraction (PAF) estimation and bootstrap-based Monte Carlo uncertainty analysis.

### 2.2. Study Population and Analytic Sample

The initial 2019 dataset included 5603 participants. Participants younger than 18 years were excluded from the analysis (n = 124), resulting in an adult analytical source population of 5479 participants. Among these, 54 participants had missing core survey information and were excluded before survey-weighted analyses, leaving 5425 participants in the eligible survey-weighted sample.

Self-perceived oral-health status was available for 5384 participants, who formed the outcome-defined sample used for the main descriptive comparison of oral-health status. For analyses involving the combined nutritional-risk score, participants were required to have complete data for all six nutritional-risk components. After excluding participants with missing information on at least one nutritional-risk component, 5288 participants were included in analyses involving the combined nutritional-risk score and categorical nutritional-risk variable. The fully adjusted nutrition-risk regression model and the main PAF analysis were based on 5146 complete cases after excluding participants with missing covariate data. Individual nutritional-variable analyses had slightly different sample sizes because of variable-specific item-level missingness.

### 2.3. Outcome Definition

The primary outcome was poor self-perceived oral health. This binary variable was derived from the original self-perceived oral-health variable, which classified participants into three categories: average, good, and bad oral health. For the primary analysis, participants reporting bad self-perceived oral health were coded as having poor oral health, while participants reporting average or good oral health were coded as the reference group. This dichotomization was selected to identify participants who provided an explicitly unfavorable assessment of their oral health and to distinguish them from participants who did not report their oral health as bad. Combining the average and good categories also provided a directly interpretable binary outcome for logistic regression. The classification was not selected through outcome-based optimization or statistical significance testing.

Self-perceived oral health was treated as a broad, multidimensional indicator of overall perceived oral status rather than as a measure of any single oral disease. It may reflect experiences related to dental caries, periodontal conditions, tooth loss, prosthetic problems, xerostomia, oral pain, esthetic concerns, halitosis, and other aspects of oral health, whose individual contributions could not be disentangled using this survey item.

Participants with missing self-perceived oral-health data were excluded from outcome-specific analyses. Self-perceived oral health was selected as the primary outcome because it captures the perceived burden of oral-health problems at the population level and is relevant for assessing oral-health inequalities in survey-based epidemiological studies.

### 2.4. Nutritional Exposures and Nutritional-Risk Score

Several nutritional indicators were derived from the EHIS dietary variables. Each indicator was recoded into a binary exposure variable to allow interpretable public-health comparisons and to support counterfactual PAF estimation.

Fruit consumption was classified as daily fruit consumption versus less than daily fruit consumption. Participants who reported rarely/never or weekly fruit consumption were coded as having low fruit consumption. Vegetable consumption was classified similarly, with less than daily vegetable consumption coded as an unfavorable nutritional indicator.

Sugary soft drink consumption was dichotomized as rarely/never versus weekly/daily consumption. Sweets consumption was classified as occasional or less than daily versus daily sweets consumption. Processed meat consumption was classified as rare/weekly versus two or more servings per week. Water intake was dichotomized as more than 1.5 L/day versus less than 1.5 L/day, with the latter coded as low water intake.

A combined nutritional-risk score was then constructed by summing six unfavorable nutritional indicators:

less than daily fruit consumption,
less than daily vegetable consumption,
weekly/daily sugary soft drink consumption,
daily sweets consumption,
two or more servings per week of processed meat,
less than 1.5 L/day water intake.

This composite score has not previously been validated or externally validated. The component thresholds were operationally defined using the categorical response options available in the EHIS and the conceptually unfavorable direction of each behavior. They were not selected by optimizing their associations with self-perceived oral health, using receiver operating characteristic analysis, or applying another outcome-driven procedure. Consequently, these cutoffs should not be interpreted as validated clinical or dietary-recommendation thresholds.

The nutritional-risk score ranged from 0 to 6, with higher values indicating a greater number of unfavorable nutritional factors. The score was calculated only among participants with complete data for all six components.

For regression and PAF analyses, the nutritional-risk score was used in two forms. First, a four-level categorical variable was created:

0–1 unfavorable nutritional factors,
2 unfavorable nutritional factors,
3 unfavorable nutritional factors,
4–6 unfavorable nutritional factors.

The lowest category, 0–1 unfavorable factors, was used as the reference group. Second, a binary high nutritional-risk variable was created, defined as three or more unfavorable nutritional factors versus fewer than three unfavorable nutritional factors. The grouping of the total score into 0–1, 2, 3, and 4–6 unfavorable factors, and the binary threshold of three or more factors, was informed by the observed score distribution to maintain interpretable categories and avoid sparse groups at the extremes. These thresholds were not derived by optimizing prediction of the oral-health outcome.

### 2.5. Covariates

Covariates were selected a priori based on epidemiological relevance and their potential role as confounders in the association between nutritional behaviors and oral health. The main adjusted models included gender, age group, education, employment, area of residence, region, financial status, BMI category, smoking status, and alcohol consumption.

Age was categorized into three adult groups: 18–34 years, 35–64 years, and 65 years or older. Education was categorized into primary, secondary, and tertiary education. Employment was included as a binary variable. Financial status was categorized as average, good, or bad. BMI was categorized as normal, overweight, or obese. Smoking status was categorized as never smoker, former smoker, or current smoker. Alcohol consumption was included as a categorical variable with five categories: abstinent, less than once a month, 1–3 times a month, 1–4 times a week, and daily or almost daily alcohol consumption. Area of residence was included as a binary variable distinguishing rural and urban residence, while geographical region was included according to NUTS2 classification with seven categories: Central Hungary, Southern Great Plain, Southern Transdanubia, Northern Great Plain, Central Transdanubia, Northern Hungary, and Western Transdanubia.

Self-perceived general health was not included in the primary adjusted model because it may conceptually overlap with self-perceived oral health and may partly capture the same subjective health perception underlying the outcome. However, to test whether the nutritional-risk association persisted after accounting for broader perceived health status, an additional sensitivity model was fitted with further adjustment for self-perceived general health.

Diabetes was available only as a broad self-reported indicator, without differentiation between type 1 and type 2 diabetes or information on duration, severity, treatment, or glycaemic control, and its temporal position relative to diet and oral health could not be established in this cross-sectional analysis. It was therefore not retained in the final adjustment set, particularly because its inclusion worsened overall model fit and could result in overadjustment; nevertheless, residual confounding by diabetes cannot be excluded.

### 2.6. Descriptive and Bivariate Analyses

Descriptive analyses were used to summarize the study population according to self-perceived oral-health status. Sociodemographic, lifestyle, and health-related characteristics were compared between participants with average/good oral health and those with bad oral health. Nutritional characteristics were then compared across the same oral-health groups.

Results were reported as unweighted counts and survey-weighted percentages calculated within each self-perceived oral-health group. Unweighted counts were included to show the actual number of respondents contributing to each estimate, while weighted percentages were used to represent the population distribution. Group differences were assessed using design-adjusted Pearson chi-squared tests.

### 2.7. Survey-Weighted Regression Analysis

Survey-weighted logistic regression models were used to estimate associations between nutritional-risk indicators and poor self-perceived oral health. The dependent variable was bad self-perceived oral health versus average/good self-perceived oral health. Results were presented as odds ratios (ORs), 95% confidence intervals (CIs), and *p*-values.

Three main nutritional exposure specifications were evaluated. First, the categorical nutritional-risk variable was modeled using 0–1 unfavorable nutritional factors as the reference category. Second, low water intake was modeled as an individual nutritional exposure. Third, high nutritional risk was modeled as a binary exposure, defined as three or more unfavorable nutritional factors.

Crude models were first fitted for each nutritional exposure. Adjusted models were then fitted controlling for gender, age group, education, employment, area of residence, region, financial status, BMI category, smoking status, and alcohol consumption. The primary regression model used the categorical nutritional-risk exposure. Low water intake and binary high nutritional risk were evaluated as additional exposure specifications. A sensitivity model additionally adjusted for self-perceived general health to evaluate whether the main nutritional-risk association remained after accounting for broader subjective health status.

### 2.8. Counterfactual Population-Attributable Fraction Estimation

Population-attributable fractions were estimated using model-based counterfactual predicted probabilities. This approach compared the predicted prevalence of poor self-perceived oral health under the observed exposure distribution with the predicted prevalence under a hypothetical counterfactual exposure distribution.

For each PAF scenario, a probability-weighted logistic regression model was fitted. The observed predicted prevalence was calculated using the fitted model and the original exposure distribution. The counterfactual predicted prevalence was then estimated by setting the exposure of interest to the reference or lowest-risk category while keeping the distribution of all other covariates unchanged. The PAF was calculated as:

$$PAF=\frac{P_{observed}-P_{counterfactual}}{P_{observed}}$$

where $P_{observed}$ represents the model-predicted prevalence of poor self-perceived oral health under the observed data structure, and $P_{counterfactual}$ represents the model-predicted prevalence under the hypothetical counterfactual exposure scenario.

The main counterfactual scenario shifted all participants to the lowest nutritional-risk category, defined as 0–1 unfavorable nutritional factors. Two additional counterfactual scenarios were evaluated: removal of high nutritional risk, defined as three or more unfavorable nutritional factors, and removal of low water intake, defined as shifting all participants to the adequate water-intake category. Because the data were cross-sectional, these PAF estimates were interpreted as model-based population-level burdens associated with nutritional-risk patterns rather than definitive causal effects.

### 2.9. Monte Carlo Uncertainty Analysis

Bootstrap-based Monte Carlo uncertainty analysis was used to quantify uncertainty around the PAF estimates. For each counterfactual scenario, 1000 bootstrap repetitions were performed. In each repetition, a bootstrap sample was drawn from the analytical dataset, a probability-weighted logistic regression model was refitted, observed predicted prevalence was estimated, the relevant counterfactual exposure distribution was imposed, counterfactual predicted prevalence was estimated, and the PAF was recalculated.

The distribution of the simulated PAF estimates was then summarized. The median simulated PAF was reported as the central Monte Carlo estimate, while the 2.5th and 97.5th percentiles were reported as the 95% Monte Carlo uncertainty interval. This procedure was applied to the main nutritional-risk counterfactual scenario and to the two secondary scenarios involving high nutritional risk and low water intake.

### 2.10. Missing Data and Sensitivity Analysis

A complete-case approach was used for the main regression and PAF analyses. Participants with missing self-perceived oral-health outcome data were excluded from outcome-specific analyses. The combined nutritional-risk score was calculated only for participants with complete data for all six nutritional-risk components. Fully adjusted regression and PAF models further required complete data on the included covariates.

Because individual nutritional indicators had different levels of item-level missingness, sample sizes differed slightly across descriptive nutritional analyses. The derivation of the analytical samples was summarized in a sample-selection flowchart. Sensitivity analysis was performed by additionally adjusting the primary nutritional-risk model for self-perceived general health. This sensitivity model was used to evaluate whether the association between nutritional-risk burden and poor self-perceived oral health persisted after accounting for overall perceived health status.

### 2.11. Statistical Software

All statistical analyses were conducted in Stata 19 [25]. Probability weights were specified using the svyset command, and survey-weighted descriptive and regression analyses were performed using Stata survey procedures. Statistical significance was assessed using two-sided tests, with *p*-values below 0.05 considered statistically significant. Results were reported as unweighted counts with weighted percentages for descriptive analyses and as ORs with 95% CIs for logistic regression analyses. PAF results were reported as percentages, together with Monte Carlo-derived uncertainty intervals.

## 3. Results

### 3.1. Study Population and Analytical Sample

The initial 2019 EHIS dataset included 5603 participants. After excluding participants younger than 18 years (n = 124), the adult analytical source population included 5479 participants. Of these, 5425 had valid survey weight and core survey information. Self-perceived oral-health status was available for 5384 participants, who formed the outcome-defined sample used for descriptive comparisons. Complete nutritional-risk score data were available for 5288 participants. The fully adjusted regression and population-attributable fraction analyses based on the categorical nutritional-risk score included 5146 complete cases. The sample-selection process is summarized in Supplementary Figure S1.

In the weighted outcome-defined sample, the prevalence of bad self-perceived oral health was approximately 19.2%, while approximately 80.8% of participants reported average or good self-perceived oral health.

### 3.2. Sociodemographic, Lifestyle, and Health Characteristics by Oral-Health Status

The weighted distributions of sociodemographic, lifestyle, and health characteristics according to self-perceived oral-health status are shown in Table 1. Gender distribution did not differ significantly between oral-health groups (*p* = 0.195). The proportion of participants aged 65 years or older was higher among those with bad oral health than among those with average/good oral health (37.46% vs. 20.62%, *p* < 0.001), while the proportion aged 18–34 years was lower (12.54% vs. 27.37%).

Educational distribution differed markedly by oral-health status (*p* < 0.001). Primary education was reported by 65.52% of participants with bad oral health compared with 33.95% of those with average/good oral health. Tertiary education was less frequent among participants with bad oral health than among those with average/good oral health (9.30% vs. 28.76%). Employment status also differed between groups: 40.21% of participants with bad oral health were employed compared with 60.75% of participants with average/good oral health (*p* < 0.001).

Financial status differed significantly by oral-health status (*p* < 0.001). Bad financial status was reported by 21.75% of participants with bad oral health compared with 9.47% of those with average/good oral health. Obesity was also more frequent among participants with bad oral health than among those with average/good oral health (29.59% vs. 22.96%, *p* < 0.001).

Smoking status showed significant differences across oral-health groups (*p* < 0.001). Current smoking was reported by 39.17% of participants with bad oral health and 24.96% of those with average/good oral health. Self-perceived general health also differed substantially between groups (*p* < 0.001): bad general health was reported by 25.48% of participants with bad oral health compared with 6.24% of participants with average/good oral health (Table 1).

### 3.3. Nutritional Characteristics by Oral-Health Status

Weighted nutritional characteristics according to self-perceived oral-health status are presented in Table 2. Within the bad oral-health group, 47.32% of participants reported less-than-daily fruit consumption, compared with 43.27% within the average/good oral-health group (*p* = 0.022). The corresponding percentages for less-than-daily vegetable consumption were 59.40% and 53.57%, respectively (*p* = 0.001).

No significant difference was observed for weekly/daily sugary soft drink consumption between participants with bad oral health and those with average/good oral health (34.80% vs. 35.73%, *p* = 0.590). Similarly, daily sweets consumption was nearly identical across the two groups (33.59% vs. 33.45%, *p* = 0.932). High processed meat consumption was slightly less frequent among participants with bad oral health than among those with average/good oral health (79.20% vs. 81.80%), with a borderline group difference (*p* = 0.054).

Low water intake was more frequent among participants with bad oral health than among those with average/good oral health (30.77% vs. 23.29%, *p* < 0.001). High nutritional risk, defined as three or more unfavorable nutritional factors, was observed in 58.64% of participants with bad oral health and 54.67% of participants with average/good oral health (*p* = 0.025). The categorical nutritional-risk distribution also differed by oral-health status (*p* = 0.047). Participants with bad oral health were less frequently in the 0–1 unfavorable-factor category than those with average/good oral health (17.76% vs. 21.65%) and more frequently in the 4–6 unfavorable-factor category (31.32% vs. 29.11%) (Table 2).

### 3.4. Crude and Adjusted Associations Between Nutritional Risk Indicators and Poor Self-Perceived Oral Health

Crude and adjusted associations between nutritional risk indicators and poor self-perceived oral health are shown in Table 3. In crude survey-weighted logistic regression, participants with three unfavorable nutritional factors had higher odds of poor self-perceived oral health compared with those with 0–1 unfavorable factors (OR = 1.30 [1.06–1.61], *p* = 0.013). A similar association was observed among participants with 4–6 unfavorable nutritional factors (OR = 1.31 [1.07–1.61], *p* = 0.010). The crude association for two unfavorable factors was weaker and did not reach conventional statistical significance (OR = 1.22 [0.98–1.51], *p* = 0.073).

After adjustment for gender, age group, education, employment, area of residence, region, financial status, BMI category, smoking status, and alcohol consumption, the nutritional-risk gradient remained evident. Compared with participants with 0–1 unfavorable nutritional factors, the adjusted odds of poor self-perceived oral health were higher among participants with three unfavorable factors (OR = 1.37 [1.09–1.73], *p* = 0.007) and those with 4–6 unfavorable factors (OR = 1.46 [1.15–1.85], *p* = 0.002). The adjusted association for two unfavorable factors remained borderline (OR = 1.25 [1.00–1.57], *p* = 0.054).

Low water intake was associated with higher odds of poor self-perceived oral health in both crude and adjusted models. In the crude model, participants consuming less than 1.5 L/day of water had higher odds of poor oral health than those consuming more than 1.5 L/day (OR = 1.46 [1.25–1.71], *p* < 0.001). This association remained after adjustment (OR = 1.38 [1.16–1.64], *p* < 0.001).

High nutritional risk, defined as three or more unfavorable nutritional factors, was also associated with poor self-perceived oral health. The crude OR was 1.18 [1.02–1.35] (*p* = 0.025), and the adjusted OR was 1.25 [1.06–1.47] (*p* = 0.008).

**Figure 1:**
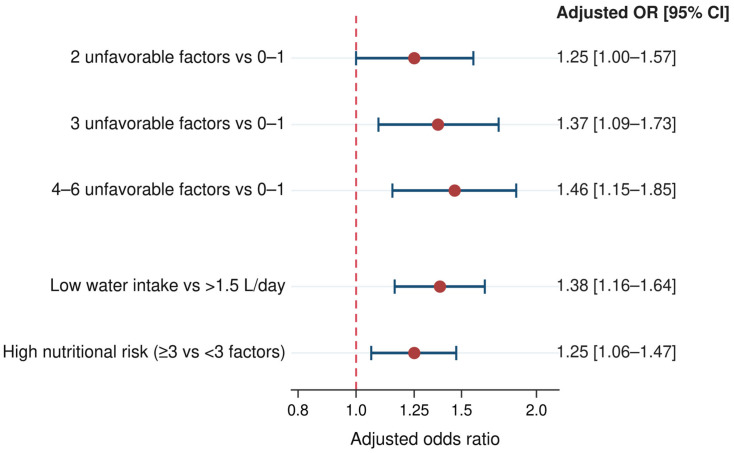
Adjusted associations between nutritional-risk indicators and poor self-perceived oral health. Footnote: Points represent adjusted odds ratios, and horizontal bars represent 95% confidence intervals. Estimates were obtained from separate survey-weighted logistic regression models for the categorical nutritional-risk score, low water intake, and binary high nutritional risk. All models were adjusted for gender, age group, education, employment, area of residence, geographical region, financial status, BMI category, smoking status, and alcohol consumption. The vertical dashed line indicates OR = 1. OR: odds ratio; CI: confidence interval.

Full adjusted models using alternative nutritional exposure specifications are provided in Supplementary Table S1. In the sensitivity model additionally adjusted for self-perceived general health, the association between nutritional-risk category and poor self-perceived oral health remained present. Compared with participants with 0–1 unfavorable nutritional factors, the adjusted OR was 1.35 [1.07–1.71] for three unfavorable factors and 1.53 [1.20–1.94] for 4–6 unfavorable factors (Table 3 and Figure 1).

Full adjusted models using the three alternative nutritional exposure specifications are presented in Supplementary Table S1. In the sensitivity analysis additionally adjusted for self-perceived general health, the association between nutritional-risk category and poor self-perceived oral health remained present, particularly among participants with three unfavorable factors and 4–6 unfavorable factors (Supplementary Table S2).

### 3.5. Counterfactual PAF and Monte Carlo Uncertainty Analysis

Counterfactual PAF estimates and Monte Carlo uncertainty intervals are presented in Table 4 and Figure 1. In the main counterfactual scenario, in which all participants were shifted to the lowest nutritional-risk category, the model-predicted prevalence of poor self-perceived oral health decreased from 19.37% to 16.26%. This corresponded to a point-estimate PAF of 16.09%.

**Figure 2:**
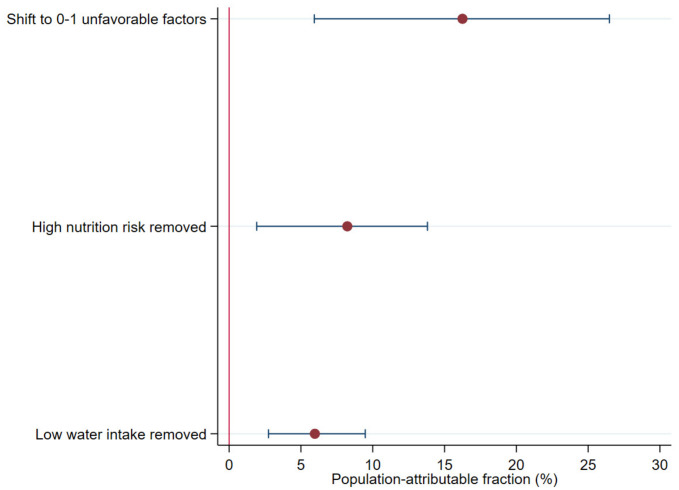
Monte Carlo-estimated population-attributable fractions for poor self-perceived oral health under alternative counterfactual nutritional scenarios. Footnote: Points represent the median simulated population-attributable fraction (PAF), and horizontal bars represent 95% Monte Carlo uncertainty intervals derived from 1000 bootstrap-based simulation repetitions. The vertical reference line indicates PAF = 0. The main counterfactual scenario shifted all participants to the lowest nutritional-risk category, defined as 0–1 unfavorable nutritional factors. PAF: population-attributable fraction.

In the bootstrap-based Monte Carlo uncertainty analysis, the median PAF for the main nutritional-risk scenario was 16.25%, with a 95% uncertainty interval of 5.93% to 26.48%. For the scenario in which high nutritional risk was removed, the point-estimate PAF was 8.22%, and the Monte Carlo median PAF was 8.23% with a 95% uncertainty interval of 1.92% to 13.81%. For the low-water-intake scenario, the point-estimate PAF was 5.93%, and the Monte Carlo median PAF was 5.97% with a 95% uncertainty interval of 2.74% to 9.48% (Table 4 and Figure 2).

### 3.6. Secondary Oral-Health Outcomes

Adjusted associations between high nutritional risk and secondary oral-health outcomes are shown in Table 5. High nutritional risk was associated with active caries (OR = 1.48 [1.28–1.72], *p* < 0.001), gum bleeding (OR = 1.50 [1.26–1.79], *p* < 0.001), and having missing teeth (OR = 1.31 [1.15–1.50], *p* < 0.001). No significant association was observed for mobile teeth (OR = 1.08 [0.85–1.36], *p* = 0.533) or difficulty eating (OR = 1.01 [0.74–1.39], *p* = 0.936) (Table 5).

## 4. Discussion

This nationally representative study of Hungarian adults found that unfavorable nutritional profiles were independently associated with poor self-perceived oral health. Participants with three or more unfavorable nutritional factors had significantly higher odds of reporting poor oral health, and a clear gradient was observed whereby increasing nutritional risk was associated with progressively worse oral-health outcomes. Furthermore, population-attributable fraction analyses suggested that approximately one-sixth of poor self-perceived oral health in the Hungarian population may be associated with adverse nutritional patterns.

These findings support the growing recognition of oral health as an integral component of the broader non-communicable disease programme and highlight the importance of dietary behaviors as potentially modifiable determinants of oral-health inequalities [26,27]. While previous studies have largely focused on individual dietary components, such as sugar consumption [28,29], the recent studies demonstrate that the cumulative burden of multiple unfavorable nutritional behaviors may be more relevant for understanding oral-health disadvantage at the population level [30,31]. The main findings of the current analysis align with this, demonstrating the associations between poor oral health and unfavorable dietary factors.

Among the individual dietary indicators examined, low vegetable consumption and inadequate water intake showed the clearest observed associations with poor self-perceived oral health. These findings are biologically plausible and consistent with emerging evidence suggesting that dietary quality influences oral health through multiple pathways beyond dental caries alone [32]. Diets rich in vegetables provide antioxidants, vitamins, minerals, and anti-inflammatory compounds that may help maintain periodontal health and support host immune responses, whereas lower consumption has been linked to a greater prevalence of periodontal disease and tooth loss in previous observational studies [33,34]. Similarly, adequate water intake may contribute to oral health through its effects on salivary flow, oral clearance mechanisms, and the maintenance of a healthy oral environment [35]. Recent epidemiological evidence has suggested that higher plain-water consumption is associated with lower odds of periodontitis, supporting the direction of the association observed in the present study [16]. Nevertheless, the association between low water intake and poor self-perceived oral health should not be interpreted as evidence of an independent causal effect. Given the cross-sectional design, low water intake may represent a marker of a broader unfavorable dietary pattern, lower health consciousness, or other clustered lifestyle and socioeconomic factors that were not fully captured by the adjusted model. Residual confounding and reverse causation also cannot be excluded. Longitudinal and intervention studies are therefore needed to determine whether increasing water intake itself produces measurable improvements in oral health.

Interestingly, sugary soft-drink consumption and daily sweets consumption were not independently associated with poor self-perceived oral health in the descriptive analyses. This finding does not necessarily contradict the extensive evidence linking free sugars to dental caries [36]. Rather, it may reflect limitations inherent in self-reported dietary measures, the broad nature of the self-perceived oral-health outcome, or the possibility that oral-health status in adults reflects the cumulative effects of long-term behaviors and social determinants [37], rather than current consumption patterns alone. In addition, oral health encompasses multiple conditions, including periodontal disease and tooth loss, whose determinants extend beyond sugar exposure [38].

An important contribution of the present study is the use of a composite nutritional-risk profile rather than the examination of isolated dietary factors. Dietary behaviors rarely occur independently, and unhealthy dietary practices often cluster within individuals. By capturing the cumulative burden of multiple unfavorable nutritional behaviors, the nutritional-risk score may better reflect real-world exposure patterns and provide a more comprehensive measure of nutrition-related oral-health risk. The observed dose-response relationship, whereby participants with increasing numbers of unfavorable nutritional factors experienced progressively higher odds of poor oral health, supports the value of this cumulative-risk approach.

The associations observed for active caries, gum bleeding, and tooth loss strengthen the interpretation that the relationship between nutritional risk and poor self-perceived oral health reflects underlying oral disease processes rather than subjective health perceptions alone. The consistency of these findings across multiple oral-health outcomes suggests that unfavorable nutritional profiles may influence oral health through several pathways simultaneously, affecting both cariogenic and periodontal conditions [39]. This observation is consistent with the concept that oral diseases share common behavioral determinants and frequently coexist within the same individuals [30]. The association observed for gum bleeding may indicate that nutritional factors are relevant not only to the development of dental caries but also to inflammatory processes affecting the periodontal tissues [40]. Dietary habits influence the oral environment, host immune responses, and broader metabolic processes, potentially contributing to the progression of oral diseases throughout the life course [41,42]. Likewise, the association with tooth loss may reflect the cumulative consequences of long-term exposure to unfavorable nutritional behaviors, given that tooth loss often represents the final outcome of chronic oral disease [43].

By contrast, the absence of significant associations with tooth mobility and difficulty eating suggests that these outcomes may be driven by factors beyond nutrition alone. Both conditions are likely influenced by a complex interplay of disease severity, age-related changes, prosthetic rehabilitation, healthcare utilization, and functional adaptation [44,45], which may decrease the contribution of nutritional factors when examined at the population level.

Diet should be considered alongside oral hygiene rather than as an isolated determinant of oral health. Both dietary exposures and oral-hygiene practices can influence the composition and activity of dental biofilms. Dietary substrates may alter microbial ecology, acid production, and the inflammatory environment, while toothbrushing and interdental cleaning affect plaque accumulation and maturation [33,40]. Their combined effects may therefore influence the development of dental caries and periodontal inflammation. With respect to periodontal disease, diet-related alterations in the oral microbiota may contribute to dysbiosis and modify local biofilm activity and the host inflammatory response. Dietary patterns may also influence the gut microbiota and its metabolites, potentially affecting systemic immune and inflammatory pathways relevant to periodontal tissues, although this gut–oral relationship remains incompletely understood [31,33,41].

At the same time, the relationship between nutrition and oral health may be bidirectional. Dental pain, tooth loss, tooth mobility, impaired masticatory function, prosthetic problems, xerostomia, and oral mucosal discomfort may alter food selection, including avoidance of hard or fibrous foods and a shift toward softer foods [37,44]. Consequently, an unfavorable nutritional profile may be partly a consequence rather than a cause of poor oral health. Because the EHIS dataset did not contain detailed oral-hygiene or microbiota data, and because the study was cross-sectional, we could not evaluate diet-oral hygiene interactions, determine whether the observed associations were mediated by changes in the oral microbiota, or establish their temporal direction.

A particularly noteworthy finding was the substantial population burden associated with unfavorable nutritional profiles. The counterfactual PAF analyses estimated that approximately 16% of poor self-perceived oral health cases could theoretically be avoided if the population had nutritional-risk profiles corresponding to the lowest-risk category. To contextualize this estimate, diet-attributable burdens for other non-communicable diseases are substantially higher. Global modeling studies suggest that suboptimal diet accounts for about 70% of new type 2 diabetes cases worldwide and 85.6% in Central and Eastern Europe, the highest burden of any region [46]. Similarly, dietary risks have been estimated to account for 34.6% of diabetes-related disability-adjusted life years (DALYs) in Europe and 25–35% of cardiovascular mortality in Central and Eastern Europe [47,48]. Against this background, a diet-attributable PAF of approximately 16% for poor self-perceived oral health remains a meaningful and policy-relevant estimate. Although oral health has rarely been included in shared-risk-factor prevention frameworks, these findings suggest that dietary improvements may contribute not only to general health promotion but also to reducing the population burden of oral-health problems. While causal inferences cannot be drawn from cross-sectional data and uncertainty remains considerable, the PAF provides a useful indication of potential population-level impact.

These results are particularly relevant in the context of Hungary, where oral diseases remain highly prevalent, and substantial socioeconomic inequalities in oral health persist [7,8]. The observed associations between nutritional risk and oral health support contemporary public-health frameworks that recognize common risk factors across non-communicable diseases. Dietary improvement strategies aimed at increasing fruit and vegetable consumption, promoting water as the preferred beverage, and encouraging healthier overall dietary patterns may therefore yield benefits that extend beyond metabolic and cardiovascular health to include oral-health outcomes. This supports the view that integrating oral health considerations into nutrition and chronic disease prevention policies may offer an efficient approach to tackling multiple health challenges at the same time [49].

Several strengths of this study should be acknowledged. The analysis was based on a large nationally representative sample of Hungarian adults and incorporated survey weights to improve population-level generalizability. The use of a composite nutritional-risk score allowed assessment of cumulative dietary risk rather than isolated nutritional exposures, while the application of counterfactual population-attributable fraction methodology and Monte Carlo uncertainty analysis provided policy-relevant estimates of population burden. In addition, the consistency of findings across the primary outcome and several secondary oral-health outcomes strengthens confidence in the observed associations.

The study also has limitations. First, the cross-sectional design precludes conclusions regarding causality or the temporal direction of the observed associations. Poor oral health may influence dietary choices, resulting in potential reverse causation. The composite nutritional-risk score was developed specifically for this analysis and has not undergone external validation. Its components were equally weighted, although their relative contributions to oral health may differ, and the selected operational thresholds may not capture the full dose-response relationships of the underlying dietary behaviors. The findings involving this score should therefore be interpreted as exploratory and require confirmation using validated dietary measures and independent populations. Second, both dietary behaviors and oral-health outcomes were self-reported and therefore subject to recall bias and misclassification. Third, residual confounding cannot be excluded. The EHIS dataset did not include detailed information on oral-hygiene practices, fluoride exposure, xerostomia, dental-visit frequency, denture-specific status, or medication classes with xerogenic potential. Although broader indicators of diabetes, medication use, last dental visit, and prosthetic dental replacement were available, they could not fully capture these specific oral-health-related exposures. Consequently, unmeasured or incompletely measured oral-health behaviors and clinical factors may partly explain the observed associations. Dichotomizing the original three-category self-perceived oral-health variable reduced the information contained in the outcome and combined participants reporting average and good oral health into a heterogeneous reference group. This operational classification may therefore have obscured differences between these categories and introduced some degree of outcome misclassification. Moreover, the dietary cutoffs were constrained by the predefined EHIS response categories rather than derived from established nutritional recommendations. In particular, the thresholds of 1.5 L/day for water intake and two servings per week for processed meat consumption represent operational categorizations rather than validated biological or clinical risk thresholds. Dichotomization may have reduced information, introduced exposure misclassification, and produced results that could differ under alternative cutoffs.

Finally, self-perceived oral health is a broad, multidimensional construct that may integrate experiences related to dental caries, periodontal conditions, tooth loss, prosthetic problems, xerostomia, oral pain, esthetic concerns, halitosis, and other aspects of oral health. Nutrition may be related to these underlying conditions through distinct biological and behavioral pathways, which could not be disentangled using the single global outcome measure. Accordingly, the primary association should be interpreted as relating to overall perceived oral health rather than to any specific oral disease, and self-perceived oral health does not replace comprehensive clinical assessment.

Despite these limitations, the findings provide evidence that unfavorable nutritional profiles are associated with poorer oral health among Hungarian adults and suggest that nutrition-related interventions may contribute to reducing oral-health inequalities and the population burden of oral disease. Longitudinal studies are warranted to clarify causal pathways and to determine whether improvements in dietary behaviors translate into measurable improvements in oral-health outcomes over time.

## 5. Conclusions

In this nationally representative study of Hungarian adults, higher nutritional risk was independently associated with poor self-perceived oral health and several adverse oral-health outcomes, including active caries, gum bleeding, and tooth loss. A clear dose-response relationship was observed, with increasing numbers of unfavorable dietary factors corresponding to progressively higher odds of poor oral health. The counterfactual PAF analyses suggested that a substantial proportion of poor oral-health burden may be linked to modifiable nutritional behaviors. These findings support the integration of dietary improvement and hydration promotion into oral-health prevention strategies and highlight the potential public-health benefits of addressing nutrition as part of a broader approach to reducing oral-health inequalities in Hungary.

## Figures and Tables

**Table 1:** Weighted sociodemographic, lifestyle, and health characteristics according to self-perceived oral-health status.

Variable	Category	Average/Good Oral Health, n (Weighted Column %)	Bad Oral Health, n (Weighted Column %)	*p*-Value
Gender	Male	1919 (46.49)	547 (48.79)	0.195
	Female	2334 (53.51)	584 (51.21)	
Age group	18–34 years	983 (27.37)	111 (12.54)	**<0.001**
	35–64 years	2151 (52.01)	528 (50.00)	
	65+ years	1119 (20.62)	492 (37.46)	
Education	Primary	1558 (33.95)	755 (65.52)	**<0.001**
	Secondary	1554 (37.29)	276 (25.18)	
	Tertiary	1141 (28.76)	100 (9.30)	
Employment	Not employed	1869 (39.25)	732 (59.79)	**<0.001**
	Employed	2384 (60.75)	399 (40.21)	
Area of residence	Rural	1247 (27.00)	477 (39.71)	**<0.001**
	Urban	3006 (73.00)	654 (60.29)	
Region	Central-Hungary	1253 (31.83)	290 (26.86)	**<0.001**
	Southern Great Plain	536 (13.04)	129 (11.50)	
	Southern Transdanubia	395 (8.65)	112 (10.34)	
	Northern Great Plain	633 (14.26)	203 (16.62)	
	Central Transdanubia	482 (11.19)	112 (9.62)	
	Northern Hungary	484 (10.28)	192 (16.49)	
	Western Transdanubia	470 (10.75)	93 (8.57)	
Financial status	Average	2383 (55.78)	683 (61.10)	**<0.001**
	Good	1367 (34.76)	188 (17.15)	
	Bad	407 (9.47)	244 (21.75)	
BMI category	Normal	1717 (42.47)	398 (36.18)	**<0.001**
	Overweight	1488 (34.57)	381 (34.23)	
	Obese	1003 (22.96)	344 (29.59)	
Smoking status	Never smoker	2390 (57.00)	453 (40.11)	**<0.001**
	Former smoker	799 (18.04)	245 (20.72)	
	Current smoker	1018 (24.96)	423 (39.17)	
Alcohol consumption	abstinent	1168 (26.02)	420 (37.10)	**<0.001**
	less than once a month	1041 (24.78)	243 (21.37)	
	1–3 times a month	944 (23.15)	156 (14.24)	
	1–4 times a week	711 (18.21)	143 (13.32)	
	daily or almost daily	346 (7.85)	159 (13.96)	
Self-perceived general health	Average	1258 (26.73)	478 (41.56)	**<0.001**
	Good	2671 (67.03)	343 (32.96)	
	Bad	309 (6.24)	304 (25.48)	

Footnote: Values are presented as unweighted n and survey-weighted column percentages calculated within each self-perceived oral-health group. *p*-values were calculated using design-adjusted Pearson chi-squared tests. The outcome was dichotomized as average/good versus bad self-perceived oral health. Percentages may not sum to 100 because of rounding. Missing values were excluded from the displayed category-specific comparisons. Bold values indicate statistical significance *p* < 0.05.

**Table 2:** Weighted distribution of nutritional characteristics within self-perceived oral-health groups.

Variable	Category	Average/Good Oral Health, n (Weighted Column %)	Bad Oral Health, n (Weighted Column %)	*p*-Value
Fruit consumption	Daily fruit consumption	2472 (56.73)	611 (52.68)	**0.022**
	Less than daily fruit consumption	1755 (43.27)	511 (47.32)	
Vegetable consumption	Daily vegetable consumption	1987 (46.43)	448 (40.60)	**0.001**
	Less than daily vegetable consumption	2237 (53.57)	671 (59.40)	
Sugary soft drinks	Rarely/never	2793 (64.27)	758 (65.20)	0.59
	Weekly/daily	1417 (35.73)	364 (34.80)	
Sweets consumption	Occasional or less than daily	2827 (66.55)	754 (66.41)	0.932
	Daily sweets consumption	1399 (33.45)	370 (33.59)	
Processed meat consumption	Rare/weekly	808 (18.20)	250 (20.80)	0.054
	Two or more servings/week	3422 (81.80)	874 (79.20)	
Water intake	More than 1.5 L/day	3241 (76.71)	788 (69.23)	**<0.001**
	Less than 1.5 L/day	994 (23.29)	338 (30.77)	
High nutrition risk	Fewer than 3 unfavorable factors	1974 (45.33)	476 (41.36)	**0.025**
	3 or more unfavorable factors	2202 (54.67)	636 (58.64)	
Nutrition risk category	0–1 unfavorable factors	965 (21.65)	207 (17.76)	**0.047**
	2 unfavorable factors	1009 (23.68)	269 (23.60)	
	3 unfavorable factors	1036 (25.56)	314 (27.32)	
	4–6 unfavorable factors	1166 (29.11)	322 (31.32)	

Footnote: Values are presented as unweighted counts and survey-weighted column percentages. Percentages were calculated within each self-perceived oral-health group; therefore, for each nutritional variable, the category percentages sum to approximately 100% within each oral-health column. *p*-values were calculated using design-adjusted Pearson chi-squared tests comparing the distributions between oral-health groups. High nutritional risk was defined as the presence of three or more unfavorable nutritional factors. The nutritional-risk category was based on the total number of unfavorable nutritional factors: less than daily fruit consumption, less than daily vegetable consumption, weekly/daily sugary soft drink consumption, daily sweets consumption, two or more servings/week of processed meat, and less than 1.5 L/day water intake. Percentages may not sum to 100 because of rounding. Missing values were excluded from the displayed category-specific comparisons. Bold values indicate statistical significance *p* < 0.05.

**Table 3:** Focused regression table for crude and adjusted associations between nutritional risk indicators and poor self-perceived oral health.

Variable	Category	Crude OR [95% CI]	*p*-Value	Adjusted OR [95% CI]	*p*-Value
Nutrition risk category	0–1 unfavorable factors	Ref.		Ref.	
	2 unfavorable factors	1.22 [0.98–1.51]	0.073	1.25 [1.00–1.57]	0.054
	3 unfavorable factors	**1.30 [1.06–1.61]**	**0.013**	**1.37 [1.09–1.73]**	**0.007**
	4–6 unfavorable factors	**1.31 [1.07–1.61]**	**0.01**	**1.46 [1.15–1.85]**	**0.002**
Water intake	More than 1.5 L/day	Ref.		Ref.	
	Less than 1.5 L/day	**1.46 [1.25–1.71]**	**<0.001**	**1.38 [1.16–1.64]**	**<0.001**
High nutrition risk	Fewer than 3 unfavorable factors	Ref.		Ref.	
	3 or more unfavorable factors	**1.18 [1.02–1.35]**	**0.025**	**1.25 [1.06–1.47]**	**0.008**

Footnote: Adjusted models were controlled for gender, age group, education, employment, area of residence, region, financial status, BMI category, smoking status, and alcohol consumption. OR: odds ratio; CI: confidence interval. Bold values indicate statistical significance *p* < 0.05.

**Table 4:** Monte Carlo-estimated population-attributable fractions for poor self-perceived oral health.

Counterfactual Scenario	Point-Estimate PAF (%)	Monte Carlo Median PAF (%)	95% Monte Carlo UI (%)
Shift everyone to 0–1 unfavorable nutrition factors	16.09	16.25	5.93–26.48
Remove high nutrition risk, ≥3 unfavorable factors	8.22	8.23	1.92–13.81
Remove low water intake	5.93	5.97	2.74–9.48

Footnote: PAF estimates were derived from counterfactual predicted probabilities using probability-weighted logistic regression. Monte Carlo uncertainty intervals were estimated using 1000 bootstrap-based simulation repetitions. PAF: population-attributable fraction; UI: uncertainty interval.

**Table 5:** Adjusted associations between high nutritional risk and secondary oral-health outcomes.

Secondary Oral-Health Outcome	Model Type	Analytical n	Missing n	Exposure Category	Adjusted OR [95% CI]	*p*-Value
Active caries	Logistic regression	4931	548	3 or more unfavorable nutritional factors	1.48 [1.28–1.72]	**<0.001**
Gum bleeding	Logistic regression	5039	440	3 or more unfavorable nutritional factors	1.50 [1.26–1.79]	**<0.001**
Mobile teeth	Logistic regression	5052	427	3 or more unfavorable nutritional factors	1.08 [0.85–1.36]	0.533
Teeth missing	Logistic regression	5073	406	3 or more unfavorable nutritional factors	1.31 [1.15–1.50]	**<0.001**
Difficulty eating	Logistic regression	5159	320	3 or more unfavorable nutritional factors	1.01 [0.74–1.39]	0.936

Footnote: The reference category for the exposure was fewer than three unfavorable nutritional factors. Logistic regression was used for all secondary oral-health outcomes. All models were survey-weighted and adjusted for gender, age group, education, employment, area of residence, region, financial status, BMI category, smoking status, and alcohol consumption. Analytical n refers to the model-specific complete-case sample. Missing n was calculated relative to the adult analytical sample of 5479 participants. OR: odds ratio; CI: confidence interval. Bold values indicate statistical significance *p* < 0.05.

## Data Availability

The data analyzed in this study are subject to the following licenses/restrictions: The data presented in this study are available upon request from Hungarian Central Statistical Office, which performed and supervised the data collection. Requests to access these datasets should be directed to the Hungarian Central Statistical Office, www.ksh.hu/?lang=en (accessed on 14 February 2026).

## Supplementary Materials

The following supporting information can be downloaded at: https://www.mdpi.com/article/10.3390/nu18152408/s1, Supplementary Table S1: Full survey-weighted logistic regression models for poor self-perceived oral health using alternative nutritional exposure specifications; Supplementary Table S2: Sensitivity analysis additionally adjusted for self-perceived general health; Supplementary Figure S1: Sample-selection flowchart for the EHIS 2019 oral-health and nutritional-risk analysis.
