# Supplementary material for: Nutritional Risk Profiles and Poor Self-Perceived Oral Health in Hungarian Adults: A Population-Based Study with Monte Carlo Population-Attributable Fraction Estimation
Source: Nutrients. 2026 Jul 23;18(15):2408. doi: 10.3390/nu18152408 (PMC13468458; doi:10.3390/nu18152408)
Supplement: Supplementary file 1 [file nutrients-18-02408-s001.zip › nutrients-4404891-supplementary.pdf]

Supplementary Table S1. Full survey-weighted logistic regression models for poor self-perceived oral health using alternative nutritional exposure specifications

Variable	Category	Nutrition risk category model OR [95% CI]	p-value	Low water model OR [95% CI]	p-value	High nutrition risk model OR [95% CI]	p-value
Nutrition risk category	0-1 unfavorable factors	Ref.		Not included	—	Not included	—
	2 unfavorable factors	1.25 [1.00-1.57]	0.054	Not included	—	Not included	—
	3 unfavorable factors	1.37 [1.09-1.73]	0.007	Not included	—	Not included	—
	4-6 unfavorable factors	1.46 [1.15-1.85]	0.002	Not included	—	Not included	—
Water intake	More than 1.5 L/day	Not included	—	Ref.		Not included	—
	Less than 1.5 L/day	Not included	—	1.38 [1.16-1.64]	<0.001	Not included	—
High nutrition risk	Fewer than 3 unfavorable factors	Not included	—	Not included	—	Ref.	
	3 or more unfavorable factors	Not included	—	Not included	—	1.25 [1.06-1.47]	0.008
Gender	Male	Ref.		Ref.		Ref.	
	Female	0.90 [0.75-1.07]	0.233	0.84 [0.71-1.01]	0.06	0.89 [0.75-1.06]	0.206
Age group	18-34 years	Ref.		Ref.		Ref.	
	35-64 years	2.26 [1.73-2.94]	<0.001	2.17 [1.67-2.83]	<0.001	2.24 [1.72-2.91]	<0.001
	65+ years	3.38 [2.54-4.51]	<0.001	3.09 [2.32-4.13]	<0.001	3.31 [2.48-4.41]	<0.001
Education	Primary	Ref.		Ref.		Ref.	
	Secondary	0.50 [0.41-0.60]	<0.001	0.49 [0.41-0.59]	<0.001	0.49 [0.41-0.59]	<0.001
	Tertiary	0.33 [0.25-0.42]	<0.001	0.31 [0.24-0.40]	<0.001	0.32 [0.25-0.42]	<0.001
Employment	Not employed	Ref.		Ref.		Ref.	
	Employed	0.61 [0.49-0.76]	<0.001	0.63 [0.51-0.78]	<0.001	0.61 [0.49-0.76]	<0.001
Area of residence	Rural	Ref.		Ref.		Ref.	
	Urban	0.74 [0.62-0.87]	<0.001	0.73 [0.62-0.86]	<0.001	0.74 [0.62-0.87]	<0.001
Region	Central-Hungary	Ref.		Ref.		Ref.	
	Southern Great Plain	0.73 [0.56-0.95]	0.02	0.71 [0.54-0.92]	0.009	0.73 [0.56-0.94]	0.016
	Southern Transdanubia	1.01 [0.75-1.35]	0.946	1.00 [0.75-1.34]	0.974	1.01 [0.75-1.35]	0.97
	Northern Great Plain	1.00 [0.79-1.27]	0.993	0.98 [0.78-1.25]	0.889	1.00 [0.79-1.27]	0.999
	Central Transdanubia	0.72 [0.54-0.95]	0.021	0.70 [0.53-0.93]	0.013	0.71 [0.54-0.95]	0.019
	Northern Hungary	1.22 [0.94-1.58]	0.139	1.22 [0.94-1.58]	0.126	1.22 [0.94-1.58]	0.138
	Western Transdanubia	0.75 [0.55-1.03]	0.074	0.71 [0.52-0.97]	0.033	0.75 [0.55-1.02]	0.065
Financial status	Average	Ref.		Ref.		Ref.	
	Good	0.64 [0.52-0.78]	<0.001	0.63 [0.52-0.77]	<0.001	0.63 [0.52-0.77]	<0.001
	Bad	1.60 [1.30-1.98]	<0.001	1.60 [1.30-1.97]	<0.001	1.61 [1.30-1.98]	<0.001
BMI category	Normal	Ref.		Ref.		Ref.	
	Overweight	0.96 [0.80-1.16]	0.708	0.97 [0.80-1.17]	0.756	0.96 [0.79-1.16]	0.65
	Obese	1.15 [0.95-1.39]	0.159	1.15 [0.95-1.39]	0.155	1.14 [0.94-1.38]	0.184
Smoking status	Never smoker	Ref.		Ref.		Ref.	
	Former smoker	1.41 [1.14-1.74]	0.001	1.40 [1.14-1.73]	0.002	1.40 [1.13-1.73]	0.002
	Current smoker	2.15 [1.78-2.59]	<0.001	2.14 [1.78-2.57]	<0.001	2.15 [1.78-2.59]	<0.001
Alcohol consumption	abstinent	Ref.		Ref.		Ref.	
	less than once a month	0.82 [0.66-1.01]	0.068	0.81 [0.66-1.00]	0.054	0.82 [0.67-1.02]	0.07
	1-3 times a month	0.68 [0.53-0.87]	0.002	0.68 [0.54-0.87]	0.002	0.68 [0.53-0.87]	0.002
	1-4 times a week	0.80 [0.61-1.04]	0.094	0.79 [0.60-1.03]	0.081	0.80 [0.61-1.04]	0.099
	daily or almost daily	1.27 [0.96-1.67]	0.099	1.23 [0.93-1.62]	0.15	1.27 [0.96-1.68]	0.098

Footnote: The outcome was bad self-perceived oral health versus average/good self-perceived oral health. Three alternative nutritional exposure specifications were evaluated: categorical nutritional-risk score, low water intake, and binary high nutritional risk. All models were survey-weighted and adjusted for gender, age group, education, employment, area of residence, geographical region, financial status, BMI category, smoking status, and alcohol consumption. Odds ratios and 95% confidence intervals are presented. OR: odds ratio; CI: confidence interval; Ref.: reference category.

Supplementary Table S2. Sensitivity analysis additionally adjusted for self-perceived general health

Variable	Category	Conservative model OR [95% CI]	p-value
Nutrition risk category	0-1 unfavorable factors	Ref.	
	2 unfavorable factors	1.23 [0.97-1.54]	0.081
	3 unfavorable factors	**1.35 [1.07-1.71]**	**0.011**
	4-6 unfavorable factors	**1.53 [1.20-1.94]**	**<0.001**
Gender	Male	Ref.	
	Female	0.88 [0.73-1.05]	0.16
Age group	18-34 years	Ref.	
	35-64 years	**1.74 [1.33-2.28]**	**<0.001**
	65+ years	**2.21 [1.63-3.00]**	**<0.001**
Education	Primary	Ref.	
	Secondary	**0.57 [0.48-0.69]**	**<0.001**
	Tertiary	**0.37 [0.28-0.49]**	**<0.001**
Employment	Not employed	Ref.	
	Employed	**0.73 [0.59-0.91]**	**0.005**
Area of residence	Rural	Ref.	
	Urban	**0.75 [0.63-0.89]**	**0.001**
Region	Central-Hungary	Ref.	
	Southern Great Plain	**0.74 [0.56-0.97]**	**0.027**
	Southern Transdanubia	1.09 [0.81-1.46]	0.579
	Northern Great Plain	0.99 [0.78-1.26]	0.951
	Central Transdanubia	0.76 [0.57-1.02]	0.063
	Northern Hungary	1.21 [0.92-1.59]	0.167
	Western Transdanubia	0.81 [0.59-1.12]	0.203
Financial status	Average	Ref.	
	Good	**0.71 [0.58-0.87]**	**0.001**
	Bad	**1.35 [1.08-1.69]**	**0.008**
BMI category	Normal	Ref.	
	Overweight	0.94 [0.78-1.14]	0.538
	Obese	1.05 [0.86-1.28]	0.632
Smoking status	Never smoker	Ref.	
	Former smoker	**1.40 [1.13-1.73]**	**0.002**
	Current smoker	**2.16 [1.78-2.61]**	**<0.001**
Alcohol consumption	abstinent	Ref.	

	less than once a month	0.91 [0.73-1.14]	0.41
	1-3 times a month	**0.74 [0.57-0.94]**	**0.016**
	1-4 times a week	0.89 [0.68-1.18]	0.415
	daily or almost daily	**1.44 [1.08-1.92]**	**0.012**
Self-perceived general health	Average	Ref.	
	Good	**0.49 [0.40-0.60]**	**<0.001**
	Bad	**1.96 [1.57-2.46]**	**<0.001**

Footnote: The outcome was bad self-perceived oral health versus average/good self-perceived oral health. The model was survey-weighted and adjusted for gender, age group, education, employment, area of residence, geographical region, financial status, BMI category, smoking status, alcohol consumption, and self-perceived general health. This sensitivity model was used to evaluate whether the association between nutritional-risk category and poor self-perceived oral health remained after additional adjustment for overall perceived health status. Odds ratios and 95% confidence intervals are presented. OR: odds ratio; CI: confidence interval; Ref.: reference category.

Supplementary Figure S1. Sample-selection flowchart for the EHIS 2019 oral-health and nutritional-risk analysis

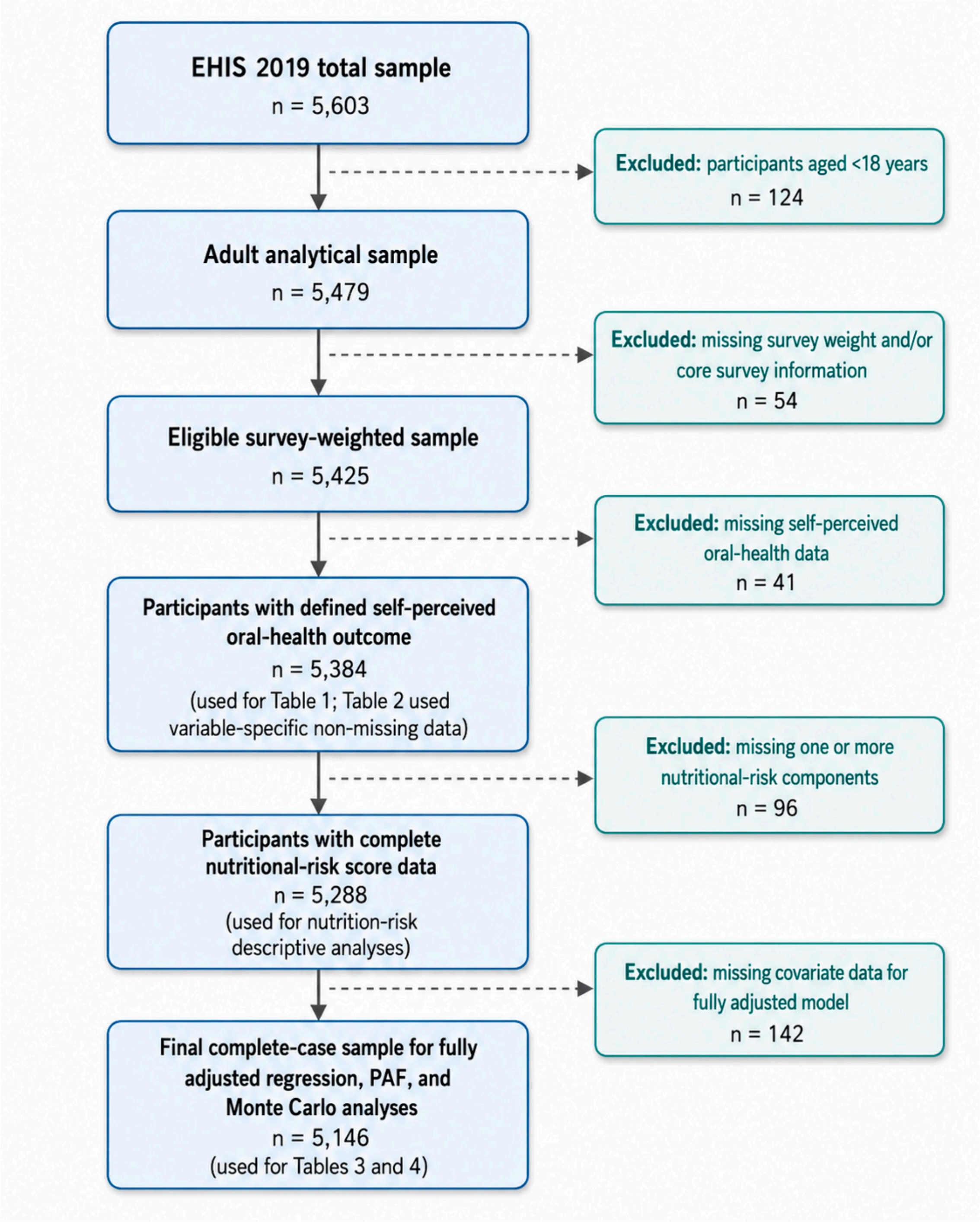

Footnote: The flowchart shows the derivation of the analytical samples from the initial EHIS 2019 total sample. Participants younger than 18 years were excluded before defining the adult analytical sample. Further exclusions were based on missing survey weight/core survey information, missing self-perceived oral-health outcome data, missing nutritional-risk components, and missing covariate data required for the fully adjusted regression, PAF, and

Monte Carlo analyses. EHIS: European Health Interview Survey; PAF: population-attributable fraction.
